# From clinical specimens to human cancer preclinical models—a journey the NCI‐cell line database—25 years later

**DOI:** 10.1002/jcb.29564

**Published:** 2019-12-05

**Authors:** James L. Mulshine, Peter Ujhazy, Melissa Antman, Christine M. Burgess, Igor Kuzmin, Paul A. Bunn, Bruce E. Johnson, Jack A. Roth, Harvey I. Pass, Sheila M. Ross, Carolyn R. Aldige, Ignacio I. Wistuba, John D Minna

**Affiliations:** ^1^ Center for Healthy Aging, Department of Internal Medicine Rush University Chicago Illinois; ^2^ Translational Research Program, Division of Cancer Treatment and Diagnosis National Cancer Institute Rockville Maryland; ^3^ Center for Research Strategy National Cancer Institute Bethesda Maryland; ^4^ Research Analytics Digital Science Cambridge Massachusetts; ^5^ University of Colorado Cancer Center University of Colorado Cancer Center Aurora Colorado; ^6^ Department of Medical Oncology Dana‐Farber Cancer Institute Boston Massachusetts; ^7^ Department of Thoracic and Cardiovascular Surgery, Division of Surgery The University of Texas MD Anderson Cancer Center Houston Texas; ^8^ Department of Cardiothoracic Surgery New York University Langone Medical Center New York New York; ^9^ Advocacy Lung Cancer Alliance Annapolis Maryland; ^10^ Prevent Cancer Foundation Alexandria Virginia; ^11^ Department of Translational Molecular Pathology UT MD Anderson Cancer Center Houston Texas; ^12^ Nancy B. and Jake L. Hamon Center for Therapeutic Oncology Research UT Southwestern Medical Center Dallas Texas; ^13^ Member IASLC Early Detection and Screening Committee Aurora Colorado

**Keywords:** adult T‐cell Leukemia‐lymphoma, cell lines, human cancer preclinical models, lung cancer, mesothelioma, patient donated specimens, precision medicine

## Abstract

The intramural the National Cancer Institute (NCI) and more recently the University of Texas Southwestern Medical Center with many different collaborators comprised a complex, multi‐disciplinary team that collaborated to generated large, comprehensively annotated, cell‐line related research resources which includes associated clinical, and molecular characterization data. This material has been shared in an anonymized fashion to accelerate progress in overcoming lung cancer, the leading cause of cancer death across the world. However, this cell line collection also includes a range of other cancers derived from patient‐donated specimens that have been remarkably valuable for other types of cancer and disease research. A comprehensive analysis conducted by the NCI Center for Research Strategy of the 278 cell lines reported in the original Journal of Cellular Biochemistry Supplement, documents that these cell lines and related products have since been used in more than 14 000 grants, and 33 207 published scientific reports. This has resulted in over 1.2 million citations using at least one cell line. Many publications involve the use of more than one cell line, to understand the value of the resource collectively rather than individually; this method has resulted in 2.9 million citations. In addition, these cell lines have been linked to 422 clinical trials and cited by 4700 patents through publications. For lung cancer alone, the cell lines have been used in the research cited in the development of over 70 National Comprehensive Cancer Network clinical guidelines. Finally, it must be underscored again, that patient altruism enabled the availability of this invaluable research resource.

## Graphical abstract

Infographic showing the results of a comprehensive analysis conducted by the NCI Center for Research Strategy of the downstream use of the 278 human cancer cell lines reported in the original cell line supplement issue (1). These cell lines and related meta data were available to the research community across the world at the cost of only shipping and cell line curation. The research and care impact of this access over the ensuing twenty five years was determined for publications, citations, supporting grants, patents and linked clinical trials.

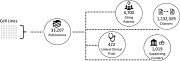

## 1 INTRODUCTION

### 1.1 Origins of lung cancer research in the NCI ‐Veteran's Administration Medical Oncology Branch and the NCI‐Navy Medical Oncology Branch

Lung cancer has been and still is the most common cause of cancer death in both men and women and in the 1970's systemic treatments were only available to patients with small cell lung cancer. Lung cancer deaths were peaking in the 1970s and 1980s based on cigarette smoking patterns. This prompted the National Cancer Institute (NCI) to develop an intramural program to study and treat patients with this deadly disease. The work was part of intramural NCI research conducted first at the NCI‐Veteran's Administration Medical Oncology Branch (NCI‐VA Branch) at the Washington, DC VA Medical Center (1975) and then at the NCI‐Navy Medical Oncology Branch (NCI‐Navy Branch) at the National Naval Medical Center (NNMC, now called the tri‐service Walter Reed National Military Medical Center) in Bethesda, MD. Both branches were established under NCI leadership efforts of the former NCI Director, Vincent DeVita. The Branches were part of the NCI's Division of Cancer Treatment's Clinical Oncology Program (Directed at that time by Samuel Broder and Gregory A. Curt). Because military veterans had a high rate of lung cancer related to a high rate of cigarette smoking and lung cancer has a high mortality rate, lung cancer was the dominant focus of the Branch's research.^1^ With the support of the leadership, initially at the Veterans Administration and later with the leaders of National Naval Medical Center, cancer care for the active and retired military, as well as relevant National Institute of Health (NIH) protocol patients, was provided under the terms of a memorandum of understanding by NIH care providers.

A major product of these studies was the generation of hundreds of lung cancer cell lines. In addition, the investigators at this government‐sponsored program were interested in the open sharing of research tools arising from this translational effort. In 1996, a supplemental issue of the Journal of Cellular Biochemistry described the cell line collection including relevant clinical, molecular, and biological data on over 300 human cancer cell lines (with a focus on lung cancer), along with 40 autologous B‐cell lymphoblastoid lines arising from some of the same patients who donated the lung cancer specimen (later this would become important because it allowed comparison of tumor and germline genomic sequences to unequivocally identify somatic mutations).^2^ The supplement included 22 research articles that included additional clinical, pathological, biochemical, and molecular characterization data. This issue was intended to be a working resource for the translational and basic research community.

Now, more than two decades after that supplement was published, there is still an avid interest in the articles contained in that publication. In light of this persistent interest, it was decided to look back over the intervening time since the Supplement to assess what was the extent of utilization of the cell line resource and further to evaluate what was the impact of those activities.

### 1.2 Lung cancer cell line generation

The lung tumor cell line generation and characterization effort were combined team efforts that involved laboratory and clinical scientists and focused on comprehensive studies of patients entered onto IRB‐approved NCI clinical trials. Thus, after informed patient consent, detailed clinical data were collected; tumor samples and other patient biosamples from multiple protocol approved staging procedures in use at the time (eg, surgical resections and biopsies of many sites) were obtained as permitted. The types of staging, imaging, biospecimen procurement procedures, and patient confidentiality issues have evolved from 1975 until the present, but the investigators used state of the art approaches for the time. The effort over a 16‐ year period (1975‐1991) involved Adi Gazdar, John Minna, Paul Bunn, Daniel Ihde, Martin Cohen, Desmond Carney, Mary Matthews, Byron Fossieck, George Higgins, Geraldine Schecter, Betty Fishman, James Mulshine, James Batty, Frank Cuttitta, Bruce Johnson, Edward Sausville, Eli Glatstein, Harvey Pass, Jack Roth, Barry Kramer, Ilona Linnoila, Marion Nau, and Stephen Veach as well as many other Navy Hospital Bethesda and NCI faculty, NCI Clinical Oncology Program fellows in training (Medical Oncology), and Navy Hospital Bethesda house officers. This multi‐disciplinary “team” included clinical and laboratory investigators, had pathologists, medical, surgical, and radiation oncologists, hematologists, dermatologists, cellular and molecular biologists, nurses, statisticians as well as cell biologists skilled in mammalian cell culture.^3^ There was outstanding support from a clinical research team of nurses and bioinformatician (Anita Johnston Early, Joyce Eddy, Maria Poblet, Ruby Phelps, Margaret Edison, Mercedes Gilliom) and laboratory research team (Edward Russell, Herbert Oie, Sylvia Stephanson). Lung cancer clinical trials were led by Marty Cohen, Daniel Ihde, Desmond Carney, Paul Bunn, James Mulshine, Bruce Johnson along with fluid collaborations with other NCI faculty including Eli Glatstein and colleagues from the intramural NCI‐Radiation Oncology Branch, as well as Jack Roth, Harvey Pass and other colleagues from the intramural NCI‐Surgery Branch. Overall clinical pathology expertize for all of the studies was provided by Mary Matthews, Ilona Linnoila, and Adi Gazdar. The diverse group of investigators evolved into a coordinated multi‐disciplinary organ system‐focused approach to translational research. Because of clinical trials focus on lung cancer, this disease was most highly represented. In addition, a series of cutaneous T cell lymphoma trials were designed and conducted by Paul Bunn, Geraldine Schecter, and Eli Glatstein. Serendipitously, clinical studies of cutaneous T Cell lymphomas and the subsequent T cell lymphoma lines and biospecimens generated from these patients led to important resources for investigating retrovirus pathology in human diseases such as the discovery of the first human retrovirus (HTLV1) and greatly facilitated discovery and work on HIV‐AIDs.^4^

An innovative strategy of the NCI‐VA and NCI‐Navy Branches was to encourage tight interprofessional interaction among all experts. This included the embedded presence of lung pathologists in all aspects of research which fostered a deep understanding of lung pathological classification within the framework of tumor biology. A more systematic approach to lung cancer research resulted in a focused exploration of lung preneoplasia and cancer histologic types from the perspective of neuroendocrine, adenomatous, and/or squamous differentiation across the spectrum from normal epithelium to metastatic disease. This disease orientation seeking to elucidate mechanistic scaffolding for cancer progression has now been clearly pursued across many international research teams but the integrated approach was novel in the 1970s and 1980s.

### 1.3 Training of the next generation of scientists

A key aspect of this study dynamic was the linkage of the Branch to the NCI intramural oncology training program which was a common initial starting point for many of the Branch faculty. An integral part of the fellowship experience was that all trainees participated in a laboratory project over the final 2 years of their fellowship. The Branch's research productivity was enhanced by a steady flow of highly motivated oncology fellows populating the research environment along with numerous visiting international clinicians and scientists who came to spend 1 to 3 years working on doctoral or post‐doctoral research projects.

In the NCI research setting, systematic experimental research was enabled by the availability of tumor‐derived cell lines. The generation of tumor cell lines permitted more extensive molecular and genomic characterization and the ability to test patient materials for sensitivity to potentially active chemotherapeutic agents and then relating those results back to the actual response of the patients to their various treatments. Many of the Branch research protocols conducted during that time included this correlative activity. While lung cancer research was the principal focus at the Branch, the approach to other diseases including cutaneous T‐cell lymphoma involved a similar strategy in the handling of the biopsied specimens. As a result, hundreds of human cell lines and related products, mostly of lung cancer origin, were derived from human clinical specimens to enable further research to improve patient outcomes for their cancers. Using the best available cell culture methods available at the time, efforts were also put into place to protect patient confidentiality for patient research volunteers.^3^, ^4^, ^5^ All patients admitted to the NCI intramural Clinical Oncology Program received care as part of research protocols in which care costs were covered by the NIH, independent of whether they agreed to provide their biopsy material for research purposes. Of note, the vast majority of the patients consented to provide their biospecimens for research. Given the reputation and travel resources of the NCI/NIH, people from all walks of life and all regions of the country were able to participate in these studies. The value of cell lines as a translational research substrate was obviously enhanced and recognized by the research community by the availability of an array of clinical, biochemical, pathological, and molecular characterization data.

## 2 RESEARCH ACTIVITIES

Research activities of the Branch performed after Institutional Review Board approval involved collecting clinical information that was attached to the patient biospecimens obtained and then for the related cell lines generated. Typically, this included age, gender, associated medical conditions, smoking history, performance status, histopathologic diagnosis, tumor stage, and prior cancer treatment. The pathologic diagnoses of tissue specimens and cytology of cellular specimens were defined by a panel of pathologists including Mary Matthews, Adi Gazdar, and Ilona Linnoila through a collegial consensus process. These studies also led them to teach and lead interactions with other pathologists around the world concerning the study of the evolving state of lung cancer histopathologic diagnosis. On a more direct teaching note, these highly regarded pathologists reviewed “at the microscope” individual patient diagnoses with the actual physicians responsible for caring for each of the patients, thus teaching nonpathologists about the methods and importance of histologic diagnoses. A team of highly engaged research nurses obtained and recorded information which was then validated by the Study Principal Investigator. Data team meetings followed presentations of each individual's case at the Branch's' weekly, multi‐disciplinary tumor board attended by all members of the research team. The novel, hierarchical data management system used at the Branch, was developed by Marc Serber, Memorial Sloan Kettering Cancer Center and through a highly productive and sustained interaction with Branch coordinated by Ruby Phelps. The data system could be flexibly deployed with predefined data elements required for a particular protocol while enabling efficient storage and reanalyses the accreted metadata.^6^ Therefore, this integrated cell line and data resource allowed a number of applications from personalized drug therapy selection within the context of clinical research studies.^7^, ^8^ In addition, the cell line data resource enabled innovative exploratory studies generating new in vitro and in vivo studies and the new results could be aggregated with the initial outcomes data presented in the original cell line supplement publication so that the data and tumor cell line resource continued to evolve and mature as a strategic translational research resource.^2^, ^9^, ^10^, ^11^, ^12^, ^13^ While all these approaches are regularly employed now, they were innovative at the time they were deployed.

### 2.1 Strategic importance of cell lines as a portal to global collaboration

Adi Gazdar established a working relationship with Robert J. Hay of the American Type Culture Collection (ATCC) in Rockville, MD to coordinate an efficient mechanism of cell line storage and dissemination by the ATCC of the hundreds of derived cell lines.^14^ A major emphasis of this relationship was employing best‐practices with accurate curation of the cell line provenance (eg, “DNA fingerprinting”, mycoplasma testing to assure the cell lines were not infected) to ensure the purity and consistency of the cell line or related product over time, practices which have been maintained and strengthened over time. While these are standard practices now, at the time this approach, pioneered by Gazdar, was a unique, and vitally important aspect of the cell line generation effort. Instituting these practices meant that major problems of cell culture (cell line mix‐up and contamination, and mycoplasma infection) which would be identified in the ensuing years for other human cell lines, were avoided or minimized. Of course, the scientific communities' knowledge of these provenance efforts, coupled with the detailed clinical annotation of the cell lines, dramatically increased their demand by the research community outside the NCI intramural program. As a result, investigators had access to accurately annotated cell lines that reflected the broad diversity of clinical characteristics of the patients whose tumors give rise to permanent cell lines. That enabled researchers to identify mechanisms driving lung cancer progression from many individuals with varied clinical backgrounds. The diversity is reflected in the variable genetic and biochemical features described in the supplement.^2^

The seminal paper of Hanahan and Weinberg crystalized the shared “hallmark” features of cancers providing a rational framework for new prevention and therapeutic approaches.^15^ Early work in the NCI‐intramural program helped to establish a fuller picture regarding the plasticity that lung cancer cell lines exhibit in adapting to exceeding stringent growth conditions.^16^, ^17^ An important aspect was the discovery the lung cancer cells could replicate in completely defined media (such as the ability of small cell lung cancer to grow and survive in “HITES” media, basal media supplemented only with small amounts of hydrocortisone, insulin, estradiol, and selenium). This led to the discovery the lung cancer cells were producing multiple growth factors. This reflected the tumor cell lines' ability to adapt to exceeding stringent growth conditions by producing their own trophic factors to directly (“autocrine” effects) and as well as tumor production of trophic factors on other cells such as those in the tumor microenvironment (“paracrine” effects). These discoveries revealed new directions for lung cancer‐directed precision therapies.^18^, ^19^, ^20^, ^21^, ^22^, ^23^, ^24^, ^25^, ^26^, ^27^, ^28^, ^29^, ^30^, ^31^, ^32^

The work of Shoemaker^33^ in using a 60‐cell line resource (involving human cancer lines of many lineages) provided another important resource for therapeutic development as it encapsulated the potential mechanistic variability associated with cancer. Results using this resource could be efficiently analyzed in innovative ways as a matrix to differentiate mechanisms of drug resistance. This resource includes cell lines from many sources but 6 of the 60 cell lines are from the intramural NCI including five of nine non‐small cell lung cancer lines; NCI‐H H226, H23, H322M, H460, H522, and the single colon cell line; HCC2998. https://dtp.cancer.gov/discovery_development/nci‐60/cell_list.htm.

An important feature of cell lines as research tools is that they can be cryopreserved, stored, and available for continuous propagation at different times when experimentally needed, and then be distributed on request to other laboratories. This allowed access to the same cell line with defined biochemical or molecular features to laboratories across the world to enable reproducible experiments to be performed, reported and then independently validated. While there was undoubtedly some “drift” in characteristics of individual tumor cell lines as they were propagated around the world, an important featured turned out to be the stability of their major oncogenotype (DNA mutations) and messenger RNA (mRNA) expression characteristics. An easy way to compare these aspects is to look at mutation and expression data in multiple large databases such as the Cell Line Encyclopedia‐CCLE, COSMIC, CellExpress, EMBL‐EBI Expression Atlas, The Human Protein Atlas, CellLineNavigator, Gene Expression Database of Normal and Tumor Tissues). For example, hundreds of labs have found the main driver mutations (eg, TP53, Rb1, KRAS, EGFR, STK11/LKB1) to remain stable in the tumor cell lines. A study comparing molecular features of 12 non‐small‐cell lung carcinoma (NSCLC) cell lines established at UTSW and corresponding primary tumor tissues demonstrated that NSCLC cell lines in the large majority of instances retain the properties of their parental tumors for lengthy culture periods, and they appear very representative of the lung cancer tumor from which they were derived.^34^ Also, if one compares mRNA expression profiles (by either microarrays or RNAseq technologies) of individual lines by many different laboratories or databases, the expression profiles are so constant that they represent a “fingerprint” of the lines, so when deviations occur, in nearly every case this represented a “mix‐up” of the cell lines rather than a change in mRNA expression profile of the cell lines. Overtime, all of the work of Branch faculty and collaborators was aggregated in the original cell line supplement.^2^ The accreted information for an individual cell line provided and continues to provide the research community with an even more valuable resource as the growing biochemical, molecular, and genomic information more precisely defines the mechanistic context of individual cancer immortalized as a cell line. Examples of large studies which use these human tumor cell lines can be found are in the Cancer Cell Line Encyclopedia (CCLE, https://portals.broadinstitute.org/ccle) “COSMIC” cell line database (Catalog of Somatic Mutations in Cancer) (https://cancer.sanger.ac.uk/cell_lines), and The EMBL Human Expression Atlas (https://www.ebi.ac.uk/gxa/experiments/E‐MTAB‐2770/Results).

### 2.2 Analyzing the impact of the patient clinical specimen donation and the resultant cell lines on research and clinical translation

To quantitatively assess the impact of the specimens donated by the NCI VA/Navy patients, we searched for publications associated with each of the 322 cell lines from the NCI/UTSW‐Cell Line Database, which were identified using full text search for the name of the cell line (such as NCI‐H460 or H460) in the Dimensions publication database (https://app.dimensions.ai/discover/publication). Full‐text search was employed as the cell lines used in an experiment may have discussed only in the methods or results section of an article and these publications would be excluded from a search based only on title and abstract. To reduce the number of false positives, the search was limited to publications that included the phrase “lung cancer” in the full text or those that had been classified as Lung Cancer using the Dimensions RCDC category scheme. In addition, the returned results were limited to articles that were classified as Cancer using either the Fields of Research or Dimensions RCDC categories. Publications were included if they were indexed in Dimensions before 30 April 2019. A total of 322 cell lines were searched, and 278 cell lines were associated with one or more publications using this method.

As a full text search was utilized, the search was likely to include articles that did not primarily employ the cell line. For example, results might have included articles that cite a publication with the cell line name in the title of the reference as well as articles that mention prior work with the cell line in the introduction. Publications are linked to a clinical trial using two methods. First, the publication can reference the clinical trial. This occurs for example when the publication includes results from the clinical trial. Second, the clinical trial can reference the publication when the trial is registered on https://clinicaltrials.gov/. An organization was considered to be a supporting funder if it was cited in the publication through the funding acknowledgment section of a publication or through other data sources such as PubMed (https://dimensions.figshare.com/articles/A_Guide_to_the_Dimensions_Data_Approach/5783094). A full table of impact by cell line is provided in Data S1 and hosted as a Database.

**Figure 1:**
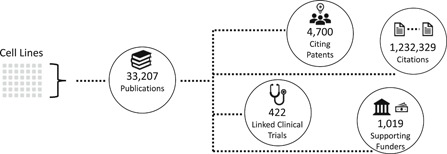
Infographic showing the results of a comprehensive analysis conducted by the NCI Center for Research Strategy of the downstream use of the 278 human cancer cell lines reported in the original cell line supplement issue. The impact of these publications includes over citations (both by publications and patents) and linked clinical trials. The number of supporting grants was also identified. NCI, National Cancer Institute

A comprehensive analysis conducted by the NCI Center for Research Strategy of the 278 cell lines reported in the original supplement identified more than 33 207 published scientific reports that have used these cell lines along with 14 057 awarded grants. The research impact of these publications is summarized in Figure 1. Notably, 38% (12 846) of these articles reference more than one cell line suggesting that researchers are often leveraging multiple cell lines in the resource to address research questions.

As an objective measure of the collective influence of the cell lines, the number of scientific outputs, such as publications that resulted from research that utilizes the cell lines is impressive. Table 1 demonstrates a sub‐analysis of the top five most productive cell lines based on the number of citing publications. The lung cancer cell line, H460 was associated with the largest number of publications (>11 000). In 50% (5663) of H460 associated publications, other cell lines further indication of the value of the resource collection. H460 was most frequently identify with H1299, H23, and H226 (found in 42%, 25%, and 19% of H460 articles respectively).

**Table 1:** The top five cell lines based on the total number of associated publications. Publications were identified through full‐text search in dimensions

Cell line	Number of publications	Number of citations	Number of patents citing publications	Number of clinical trials linked to publications	Supporting funders
H460	11 124	347 117	1564	118	717
H1299	9388	293 692	1076	71	670
H1975	3291	95 788	511	58	409
H23	2998	109 481	653	33	433
H358	2675	101 540	595	36	426

Overall for all cell lines, publications involving one or more of the cell lines have been cited over 1.2 million times. As multiple cell lines may be mentioned in an individual paper, one can also consider the sum of citations to the individual cell lines to understand the value of the resource collectively rather than individually; this method leads to 2.9 million citations. In addition, these publications have been linked to 422 clinical trials and cited by 4700 patents. Collectively these results highlight the impact that these cell lines have had on the scientific community (as indicated by the number of publications and citations) as well as on the translation of research to the clinic (as indicated by publication links to clinical trials and patent citations).

**Figure 2:**
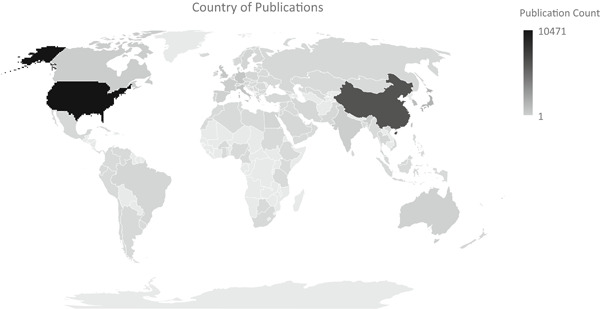
The number of unique cell‐line publications associated with each country based on the author's affiliations

The global impact of the resource can be seen in Figure 2, which displays the number and geographical distribution of unique cell‐line publications associated with each country based on the authors' affiliations. As shown schematically in Figure 2, authors from 110 countries contributed to these publications, indicating the global impact of these cell lines.

Finally, it is also important to note that upwards of 10 cell lines were developed from rare tumors including mesothelioma for which there are only 3000 cases per year in the United States. To date, there have been over 200 peer reviewed publications involving the most frequently used mesothelioma cell lines representing various tumor histologies (epitheliod, biphasic, and sarcomatoid) H28, H2052, H2373, and H2452.

### 2.3 Moving the tumor cell line effort from the NCI to the University of Texas and the establishment of a Specialized Program of Research Excellence (SPORE) in lung cancer

In 1991, John Minna and Adi Gazdar moved from the NCI to the University of Texas Southwestern (UTSW) taking the cell line collection with them. There they joined forces with the UT MD Anderson Cancer Center (MDACC) team led by Jack Roth and secured an NCI Specialized Center of Research Excellence (SPORE) grant in lung cancer. This large NCI‐driven translational program gave new opportunities for collaborations with other Lung Cancer SPOREs and lung cancer investigators in the USA and abroad. The tumor cell line repository became a central part of the SPORE Pathology Core which was co‐led by Dr. Gazdar at UTSW and Dr. Ignacio Wistuba at MDACC. The further molecular characterization of the tumor lines as part of the SPORE and their use resulted, for example, in seminal discoveries of the mutational landscape in lung cancer that would drive the development of targeted therapies representing a new era in lung cancer treatment.^35^, ^36^, ^37^, ^38^, ^39^, ^40^ More recently, the SPORE has focused on development of patient‐derived xenografts (PDXs) in efforts led by Dr. Jack Roth and which also involved a UTSW collaboration with Drs. Stephen Lam and Yuzhuo Wang of the BC Cancer Research Centre in Canada. In this effort first funded by the SPORE and subsequently by an NCI U54 PDX consortium grant, lung tumor samples were taken directly from patients and implanted in immune‐deficient mice which allowed the growth of human tumor cells. Currently, there are more than 200 such lung cancer PDXs are undergoing molecular characterization, and several of them have been used to generate cell lines. The PDXs and lung cancer cell lines as preclinical models are critical to ongoing drug development and validation studies.

### 2.4 Precision medicine targeting “oncogene addictions”: tumor cell lines in elucidating the molecular basis of EGFR mutations and ALK rearrangements in lung cancer

Elegant examples of the impact of the role of the intramural cell lines are cited with the molecular analysis on epidermal growth factor receptor (EGFR) mutations and anaplastic lymphoma kinase (ALK) rearrangements.^36^, ^39^ Two teams of researchers at the Dana‐Farber Cancer Institute and Massachusetts Hospital studied patients who had dramatic responses to the EGFR tyrosine kinase inhibitors (TKIs) and discovered mutations in the epidermal growth factor receptor. The lung cancer cell lines established at the NCI‐Navy, NCI‐H3255, and NCI‐H1975 with mutations in the tyrosine kinase domain of EGFR were able to validate that the sensitivity of cell lines to gefitinib and erlotinib was driven by these EGFR mutations.^41^, ^42^ The clinical annotation allowed the investigators led by BE Johnson and subsequent studies by investigators at other institutions to identify lung cancer cell lines which arose in women with adenocarcinoma who did not smoke, clinical characteristics associated with responses to EGFR‐TKI.^39^, ^41^, ^42^ The cell line, NCI‐3255 from such a case was sensitive to nM concentrations of gefitinib while NCI‐H1975 had both an L858R and a T790M mutation, the latter rendering it resistant to gefitinib and other (erlotinib) first‐generation EGFR TKIs. However, NCI‐H1975 was shown to have an increased response to exogenously added EGF demonstrating the biologic activity of the EGFR mutations. Subsequent work showed that the T790M mutation is the most common form of acquired resistance to EGFR‐TKIs in patients with sensitizing mutations of EGFR.^36^ This tumor line also represented “an experiment of nature” because the line was generated (and contained the T790M mutation) before EGFR TKIs were deployed in the clinic indicating the mutation arose without drug selection. This study with H1975 cells provided the in vitro molecular target to identify the pyrimidine‐based compound (osimertinib) which was active in patients with acquired resistance to EGFR‐TKIs.^36^, ^42^ Combined, this study which began and was fueled by studies on the lung tumor cell lines, led to routine testing for EGFR mutations in patients with non‐squamous NSCLC lung cancer and the Food and Drug Administration (FDA) approval of five different drugs for patients with EGFR mutations, gefitinib, erlotinib, afatinib, dacomitinib, and osimertinib. Their median survival of patients whose lung tumors have an activating EGFR mutation is prolonged by such EGFR TKI targeted therapy, and is now more than 2 years.^43^

The identification of ALK rearrangements was initially done in childhood anaplastic lymphomas.^44^ The rearranged gene was then identified in lung cancers from patients studied in Japan in 2007.^45^ These preclinical findings were rapidly translated to the clinic using cell lines derived from patients in the intramural program. Both Dr. Pasi Jαnne's team and Dr. Jeffrey Settleman's teams were able to screen lung cancer cell lines, most arising from patients treated in the intramural program and were able to identify two different cell lines, NCI‐H2228 and 3122, with ALK rearrangements which were exquisitely sensitive to the drug, crizotinib, which could target ALK.^46^, ^47^ Crizotinib was already in clinical trials starting in 2006 so an ALK‐rearranged cohort was added to the drug development protocol. The response rate of 57% in lung adenocarcinomas with ALK rearrangements and the progression‐free survival of treated patient was in excess of 6 months, and led to the FDA approval of crizotinib for ALK rearranged tumors in 2011.^48^ There are now five different FDA approved drugs, crizotinib, ceretinib, alectinib, brigatinib, and lorlatinib, for ALK‐rearranged tumors and the median progression‐free survival is nearly 3 years. This paradigm of the discovery of oncogenic drivers, screening for the sensitivity of existing and developing drugs in tumor cell lines with the appropriate genomic changes and then testing the drug in the subset of lung cancer patients with these genomic changes in now standard for drug development. This has led to FDA approved drugs for EGFR mutations, ALK rearrangements, ROS1 rearrangements, BRAF mutations, and NTRK rearrangements making up more than 20% of non‐small cell lung cancer patients.^49^

### 2.5 Further use of the lung cancer preclinical models for clinical translational research

More recently, the lung cancer cell lines were used in exploring the relationship of oncogenotypes (like TSK11/LKB1 mutations to resistance to checkpoint inhibitors) to new immunotherapies for lung cancer.^50^, ^51^ The University of Texas (UTSW/MDACC) Lung Cancer SPORE (P50CA070907), through use of these lung cancer preclinical models for its translational research, would became one of the most productive SPOREs in the 27‐year history of the entire SPORE Program with more than 1100 publications and more than 70 000 citations with an impressive weighted relative citation rate (RCR) of 2022 so far. Other NCI programs benefited from the lung cancer cell lines; the Early Detection Research Network in the development of early detection and risk assessment biomarkers and the Small Cell Lung Cancer (SCLC) NCI U24 Consortium in the exploration of the biology, molecular characterization, and search for novel therapies for one of the deadliest cancers are notable examples.

Now, 25 years after the publication of the original cell line supplement, we are seeing an unprecedented acceleration of drugs approved for precision medicine as well as general therapeutic applications in cancer, and especially in lung cancer. The direct impact of NCI cell lines on the current standard of cancer care can be also measured by screening all references in the National Cancer Comprehensive Network (NCCN) Clinical Practice Guidelines in Oncology. These guidelines are the recognized standard for clinical policy in cancer care and are the most detailed and most frequently updated clinical practice guidelines available in any area of medicine. As of March 2019, there were 71 references related to these cell lines in the NCCN Guidelines. The Guidelines are adopted by many countries and thus today hundreds of thousands of patients from all over the world benefit from the legacy of the NCI cell line work, all over the world.

### 2.6 Experience with adult T‐cell leukemia‐lymphoma and cutaneous T cell lymphoma cell lines

The NCI‐VA Medical Oncology Branch developed a number of clinical trials for patients with cutaneous T‐cell lymphomas (CTCLs) which included mycosis fungoides and the Sezary syndrome. Because the branch members were interested in growth factors that stimulated cancer growth and progression and because their cell biology models included the establishment of cultures of human cancer cells, the cells from CTCL patients were cultured. Originally, conventional techniques were used but resulted in no continuous cultures in 32 specimens from 25 patients.^52^ Subsequently, lymphocyte conditioned media was used and two cell lines with tumorigenicity in nude mice, loss of dependence on LCM and convoluted nuclear morphology were established.^53^ These cell lines were named NCI‐H78 and NCI‐H102.^33^, ^54^ Dr. Bernard Poiesz was a Clinical Oncology Program fellow in the branch who, along with Frank Ruscetti, was conducting research in the NCI laboratory of Robert Gallo exploring the role of interleukin 2 (at that time called “T Cell Growth Factor”) in T‐cell growth and maturation. One of these lines and two other cell lines from CTCL patients were subsequently found to be constitutive producers of T cell growth factor.^55^ The Gallo lab was also interested in human viruses that could cause leukemias or lymphomas so Dr. Poiesz performed a reverse transcriptase assay (at that time the most robust method of detecting retrovirus production), on supernatents from the two lines and determined that the H102 cell line had a strong signal indicating the production of a large amount of particle associated reverse transcriptase. Subsequent electron microscopic studies showed budding retrovirus termed human tumor leukemia virus 1 (HTLV1 (CR)) after the initials of the patient resulting in a seminal publication by Poiesz et al^54^, ^56^ This patient was shown to have antibodies to the virus which led to an antibody detection test to detect the virus in the blood of other patients.^57^ The ability to identify patients with the disease led to the definition of adult T‐cell leukemia/lymphoma (ATLL) and the clinical profile of patients with this disorder. These clinical features included patients from the Caribbean, southwestern Japan, and the southeastern US who had an aggressive course with stage 4, hypercalcemia, skin, and blood involvement.^58^, ^59^, ^60^ Cytogenetic studies revealed frequent deletion of chromosome 6q5.^61^ Studies from the Gallo lab indicated that another T cell lymphoma line (NCI‐H78, or “Hut 78) which did not produce HTLV1 could propagate the HIV AIDS retrovirus. While their initial publication labeled this T cell lymphoma line as “H9,” subsequent DNA fingerprinting demonstrated that this was the NCI‐H78 CTCL line which the branch had given to the Gallo lab.^62^, ^63^ This CTCL line developed as part of the tumor cell line program, thus played a central role in the early studies of HIV AIDs.

### 2.7 Patient/advocate perspective on the value of the cell‐line database and the importance of patient participation in cancer research

The NCI‐VA Branch began in the mid‐seventies, just as the public moved beyond discussing cancer in hushed voices and advocacy organizations started to emerge. More specifically, this was the beginning of cancer lineage (organ)‐specific organizations (such as breast and prostate cancer) that enabled patients to take an active and directive role, not only in their own treatment but in public health policy and research. It was during this time that donating cell lines and tumor tissue became a uniquely personal way of contributing to the presidentially mandated “War on Cancer” ‐ the popular name given to the National Cancer Act of 1971. For lung cancer patients, isolated by the sheer lethality of the disease often coupled with the burden of blame from smoking cigarettes, donating their clinical specimen for research purposes to generate a cell line was considered by many to provide a meaningful and positive legacy. While now the role of tobacco companies in manipulating nicotine content to increase addiction is public knowledge now, 25‐years ago, the majority of the newly diagnosed lung cancer patients at the NCI had already stopped smoking. We are now more aware of the stigma associated with the diagnosis of lung cancer and this great burden is even experienced by individuals with lung cancer who have never smoked. It is painful to remember that at the time of these studies that both the lay and scientific public put the “blame” for lung cancer on the patients themselves. In this environment, many patients spontaneously expressed that it gave them solace to think that in the future, others could thus be spared the devastating consequences of their cancer through their donation of clinical specimens and access to clinical data.

#### 2.7.1 Honoring donor intent

Now more than two decades after reporting the initial cell line supplement issue, we want to provide stewardship by reporting on the impact of the contribution of patients who did enroll in the relevant intramural clinical trials described in this manuscript and gave informed consent for participation in the clinical protocols and sample collection for research. Since an important motivation for many study participants was their hope that knowledge derived from the research study may help them or others in the future experience better outcomes, we are now reporting objective feedback on the impact of their donation. In addition, the commitment made to patient study subjects in Bethesda was to share all the materials and knowledge generated by the research, as broadly as possible. We are also now reporting data to reflect how widely available these patient donations were shared. From the objective analysis data in this report, it is clear that thousands of other investigators from around the world had access to the cell line and related research material, either directly from the NCI‐VA or NCI‐Navy Branches, from the large collection was deposited at the American Type Culture Collection (ATCC), or from the investigators who had worked in past at the NCI‐ Navy Branch. The initial step to address the issue of wide and equitable dissemination of the cell lines and associated data was the Cell‐line Supplement developed in collaboration with the American Type Tissue Culture Collection and the Journal of Cellular Biochemistry.^2^

For the advocacy community frustrated with the cost and slow pace of innovation, patient‐derived cell lines have an important dimension. Many aspects of conducting cancer research are expensive. Through the work of these NCI branches led by Gazdar and colleagues, hundreds of cell lines were made available at the modest cost of shipping and handling charges, which makes internationally impactful biomedical research possible. Through the Gazdar arrangement with the American Type Tissue Culture Collection, all of the carefully curated, NCI cell lines were economically, efficiently distributed to the entire international cancer research community. This aspect of the cell line legacy is indeed respectful of the broadly shared donor‐intent to maximize the public good of this patient donated material. Fortunately, this dimension of cancer research equity persists to this day and with the evolution of sub‐collections of cell lines sharing particular mutational signatures, cell lines have become an aspect of the “lingua franca” of international cancer research, especially in regard to precision medicine for lung cancer.

#### 2.7.2 Patient donations are essential to translational innovations

Patients today are more educated about their cancers and are aware that extraordinary advances have taken place in recent decades with early detection, diagnosis, and treatment of all types of cancers and diseases. However, most of society is not aware that patent‐donated specimens in general and derived cell lines in particular, along with the shared associated clinical information aggregated in open research databases have been foundational in enabling the rapid pace and the extent of these biomedical transformations, especially in lung cancer. In this regard, the impact of the altruistic gestures of previous clinical research participants has been overlooked. An important motivation for the analysis contained in this manuscript is to celebrate the importance of the many patients who so generously supported the NCI‐VA and NCI‐Navy open research efforts.

Tracking the history of these cell lines, from concept discussions at NCI in 1975, to the specific breakthroughs that underpin so many major advances emanating from them since then will establish an accurate record, recognize the foresight of Drs. Gazdar, Minna and all their translational research colleagues, and gives donor patients and the families of donor patients the satisfaction of knowing that they have indeed made an important difference in cancer innovation. This recognition reinforces the motivation for ongoing patient participation in research including the donation of their data and specimens.

#### 2.7.3 Compounding yield of NIH biomedical investment

Another important aspect of this analysis is reporting the strategic yield of the sustained NIH investment that enabled this remarkable productive global research resource. As shown in Figure 2, over 110 countries across the world have used the cell line resource in their research efforts. From a stewardship perspective, the cost of generating these several hundred cell lines in the course of already federally funded clinical trials, at a few research‐intensive sites was an incremental expense. As a result, the federally funded cell line resource to‐date has enabled over 33,000 research publications from over 14,000 research grants, that were then cited in over 1.2 million downstream scientific publications as well as in the issuing of over 4700 patents., this effort represents a profound return on taxpayer investment in NIH research.

Equally important was the NIH establishing a path for these tumor lines to be used by the pharmaceutical industry. Remember at the time the cell line project was initiated, there was not the current degree of interaction between Academia and PharmaBiotech nor the support by the Federal Government in getting NIH funded discoveries into the PharmaBiotech “pipeline” for generating new medicines. The decision by the US Government in the 1980s for such an effort was timely and expanded the impact of the NCI Branches' efforts to generate and share the tumor lines/data resource. The NIH/NCI coordinated and led the effort to widely license the tumor lines to PharmaBiotech and in so doing provided PharmaBiotech with a clear path to use the tumor lines for their discovery efforts. Without this licensing, discovery efforts would have been held back. The multi‐disciplinary team that achieved these milestones collaborated for close to fifty years. These activities involved vibrant interactions with leading researchers across the world in both industry and academia. The largely federally‐funded research focus evolved in a sustain, strategic, and scientific opportunity‐driven fashion that was then leveraged to assist targeted drug therapy efforts of PharmaBiotech. It is an open question as to whether the reported outcomes represent appropriate dividends for continuous taxpayer investment? We convey the information in this report to inform the discussion regarding the value of long‐term federal support of biomedical research.

### 2.8 Contribution of Professor Adi F. Gazdar, M.D

For the recently deceased, Adi Gazdar, establishing cancer cell lines was a means to an end. Early in his career, Adi Gazdar sought to collect sufficient cancer cell mass to perform a systematic analysis of the cancer cells for specific features. For example, having sufficient tumor cells to evaluate for in vitro drug sensitivity was an important research activity. Successfully generating a cell line to enable extensive and repetitive testing as well as sharing of the cell line so that other collaborators could interrogate the same cancer cells for their purpose. In a longtime collaboration with John Minna, this activity was continued for many decades in the intramural program of the National Cancer Institute (1975‐1991) and then moved to the University of Texas Southwest Medical Center in Dallas (1991‐2018). Over that period of time, Professor Gazdar published over 800 publications that were cited over 110 000 times including many published jointly with John Minna. Professor Gazdar mentored over 100 post‐doctoral fellows and he established an ongoing translational research fellowship award with the International Association for the Study of Lung Cancer, which he personally funded.

## 3 NEXT STEPS OF THE NCI/UTSW CELL LINE DATABASE

The tumor cell line database has expanded over the years to include generation of breast cancer, ovarian cancer, colon cancer, and rare tumor type cell lines, and a large panel of immortalized lung epithelial cell lines.^64^, ^65^, ^66^ All of these continue to be distributed on a world‐wide basis. Of importance, the molecular analyses and datasets of these cell lines have been updated to include genome wide exome DNAseq mutation and RNAseq expression analyses as well as a variety of proteomic studies including reverse phase protein arrays (RPPAs).^67^ As discussed above, many new lung cancer patient‐derived xenograft lines (PDXs) have been developed. The cell lines and PDXs can be grown in mice with a defective immune system that lacks cell‐mediated immunity and serve as an in vivo model for drug testing and from modeling drug resistance.^68^ Xenografts can be implanted in immundeficient mice transplanted with human stem cells resulting in a mouse with a human immune system growing a human tumor. This is potentially useful for evaluating new immunotherapeutic modalities such as checkpoint blockade.^69^ Large numbers of the cell lines have been used in recent, publicly available, drug response phenotype analyses, including phenotypic screening for new drugs, and functional genomics databases (such as “Project Drive” and “Dependency Map”).^70^, ^71^, ^72^ An updated version of the entire NCI/UTSW/MDACC human preclinical cell line data set with molecular genomic annotation is currently being prepared for public access.

## CONFLICT OF INTERESTS

Christine Burgess: I have the following conflicts as related to below with management plan. I am currently employed by Digital Science, the maker of the Dimensions publication database utilized in the analysis; Bruce Johnson: I receive post‐marketing royalties from Dana‐Farber for EGFR mutation testing Paid Consultant for Novartis, Hengrui Therapeutics, and Foundation Medicine Steering Committee for Novartis and Array Biopharma Research Support from Novartis and Cannon Imaging; John D. Minna: Has received cell line licensing royalties from the NCI and cell line licensing royalties from the University of Texas Southwestern Medical Center; Jack A. Roth: Consultant, stock owner (including pending patents) in Genprex, Inc.; Harvey I. Pass: Dr. Pass receives royalties from the NCI for the licensing of cell lines described in this manuscript; Ignacio Wistuba: Advisory Board: Genentech/Roche, Bayer, Bristol‐Myers Squibb, Astra Zeneca/Medimmune, Pfizer, HTG Molecular, Asuragen, Merck, GlaxoSmithKline, Guardant Health and MSD. Speaker: Medscape, MSD, Genentech/Roche, Pfizer. Research support: Genentech, Oncoplex, HTG Molecular, DepArray, Merck, Bristol‐Myers Squibb, Medimmune, Adaptive, Adaptimmune, EMD Serono, Pfizer, Takeda, Amgen, Karus, Johnson & Johnson, Bayer, Iovance, 4D, Novartis, and Akoya.

## AUTHOR CONTRIBUTIONS

Dr. Mulshine had full access to all of the data in the study and takes responsibility for the integrity of the data and the accuracy of the data analysis. All thirteen authors have all been substantially involved in the conceptualization, writing, and review of this manuscript. All have reviewed the final contents of the manuscript and accept the responsibility of authorship.

## Supporting information

Table of impact by cell line is provided and hosted as a Database
